# Supplementary material for: Foster Grandparent Programs’ Impact on the Quality-of-Life of Older Adult Volunteers
Source: Healthcare (Basel). 2025 Jan 24;13(3):230. doi: 10.3390/healthcare13030230 (PMC11817316; doi:10.3390/healthcare13030230)
Supplement: Supplementary file 1 [file healthcare-13-00230-s001.zip › healthcare-3218218-supplementary.pdf]

Table S1. Satisfaction with FGP assignment**quality of life changes.

		Satisfaction with current FGP assignment			Satisfaction with current FGP assignment
Feeling that you have purpose in life	Pearson Correlation	.229*	Sense of well-being	Pearson Correlation	.372**
	Sig. (2-tailed)	0.027		Sig. (2-tailed)	0
	N	93		N	91
Feeling you can make a positive difference in another person's life	Pearson Correlation	.278**	Physical health	Pearson Correlation	.226*
	Sig. (2-tailed)	0.007		Sig. (2-tailed)	0.03
	N	93		N	91
The amount of pleasure you gain from your daily activities	Pearson Correlation	.296**	Changes in overall quality of life	Pearson Correlation	.314**
	Sig. (2-tailed)	0.004		Sig. (2-tailed)	0.002
	N	93		N	93

*p<.05; **p<.01.
**We measure quality of life by looking at the response to questions about “purpose, positive difference, and pleasure” from the volunteering experiences with “Satisfaction of the FGP assignment.”

Table S2. Satisfaction with FGP staff**quality of life changes.

		satisfaction with the FGP staff			satisfaction with the FGP staff
Your sense of accomplishment	Pearson Correlation	.212*	The amount of pleasure you gain from your daily activities	Pearson Correlation	.320**
	Sig. (2-tailed)	0.042		Sig. (2-tailed)	0.002
	N	92		N	92
Your feeling that you have purpose in life	Pearson Correlation	.265*	Your sense of self esteem	Pearson Correlation	.297**
	Sig. (2-tailed)	0.011		Sig. (2-tailed)	0.004
	N	92		N	92
Feeling you can make a positive difference in another person's life	Pearson Correlation	.246*	Your sense of well-being	Pearson Correlation	.257*

	Sig. (2-tailed)	0.018		Sig. (2-tailed)	0.014
	N	92		N	90
Your looking forward to each new day	Pearson Correlation	.360**	Your feeling that someone is looking out for your welfare	Pearson Correlation	.294**
	Sig. (2-tailed)	0		Sig. (2-tailed)	0.005
	N	91		N	91
Your physical health	Pearson Correlation	.232*	Changes in overall quality of life	Pearson Correlation	.239*
	Sig. (2-tailed)	0.027		Sig. (2-tailed)	0.022
	N	91		N	92

*p<.05; **p<.01

**We measure quality of life by looking at the response to questions about "sense of accomplishment, purpose, positive difference, looking forward to each day, and physical health impact (self-reported) " from the volunteering experiences with "Satisfaction with the FGP staff."

Table S3. Satisfaction with volunteer site supervisor**quality of life changes.

		Satisfaction with volunteer site supervisors			Satisfaction with volunteer site supervisors
Feeling you can make a positive difference in another person's life	Pearson Correlation	.223*	Your physical health	Pearson Correlation	.241*
	Sig. (2-tailed)	0.032		Sig. (2-tailed)	0.021
	N	92		N	91
Your sense of well-being	Pearson Correlation	.285**	Your feeling that someone is looking out for your welfare	Pearson Correlation	.225*
	Sig. (2-tailed)	0.006		Sig. (2-tailed)	0.032
	N	91		N	91
Changes in overall quality of life	Pearson Correlation	0.128			
	Sig. (2-tailed)	0.222			
	N	92			

*p<.05; **p<.01

**We measure quality of life by looking at the response to questions about "positive difference, sense of well-being, and changes in overall quality of life (self-reported) " from the volunteering experiences with "Satisfaction with volunteer site supervisor."

Table S4. Satisfaction with training received**quality of life changes.

		Satisfaction with training received			Satisfaction with training received
Feeling you can make a positive difference in another person's life	Pearson Correlation	.274**	Your sense of self esteem	Pearson Correlation	.302**
	Sig. (2-tailed)	0.008		Sig. (2-tailed)	0.003
	N	92		N	92
The amount of pleasure you gain from your daily activities	Pearson Correlation	.390**	Your ability to make ends meet	Pearson Correlation	.318**
	Sig. (2-tailed)	0		Sig. (2-tailed)	0.002
	N	92		N	92
Your physical health	Pearson Correlation	.217*	Your sense of well-being	Pearson Correlation	.251*
	Sig. (2-tailed)	0.038		Sig. (2-tailed)	0.017
	N	91		N	90
Changes in overall quality of life	Pearson Correlation	.253*	Your feeling that someone is looking out for your welfare	Pearson Correlation	.235*
	Sig. (2-tailed)	0.015		Sig. (2-tailed)	0.025
	N	92		N	91

*p<.05; **p<.01.

**We measure quality of life by looking at the response to questions about "positive difference, pleasure, physical health impacts, and overall quality of life (self reported)" from the volunteering experiences to "Satisfaction with training received."

Table S5. Satisfaction with the overall experience**quality of life changes.

		Satisfaction with the overall experience			Satisfaction with the overall experience
Your sense of accomplishment	Pearson Correlation	.248*	Your looking forward to each new day	Pearson Correlation	.275**
	Sig. (2-tailed)	0.017		Sig. (2-tailed)	0.008
	N	93		N	92
Your feeling that you have purpose in life	Pearson Correlation	.312**	The amount of pleasure you gain from your daily activities	Pearson Correlation	.306**
	Sig. (2-tailed)	0.002		Sig. (2-tailed)	0.003
	N	93		N	93

Your feeling you can make a positive difference in another person's life	Pearson Correlation	.311**	Your sense of self esteem	Pearson Correlation	.359**
	Sig. (2-tailed)	0.002		Sig. (2-tailed)	0
	N	93		N	93
Your ability to make ends meet	Pearson Correlation	.310**	Your feeling that someone is looking out for your welfare	Pearson Correlation	.373**
	Sig. (2-tailed)	0.002		Sig. (2-tailed)	0
	N	93		N	92
Your sense of well-being	Pearson Correlation	.340**	Changes in overall quality of life	Pearson Correlation	.386**
	Sig. (2-tailed)	0.001		Sig. (2-tailed)	0
	N	91		N	93

*p<.05; **p<.01

**We measure quality of life by looking at the response to questions about "sense of accomplishment, purpose, positive difference, making ends meet, and sense of well-being" from the volunteering experiences to "Overall volunteering experience."

Table S6. In general, to what extent do you credit any change to your quality of your life to your participation in this program answer frequency.

		Frequency	Percent	Valid Percent	Cumulative Percent
Valid	a little responsible	1	1.1	1.1	1.1
	somewhat responsible	11	11.8	12.1	13.2
	almost fully responsible	41	44.1	45.1	58.2
	totally responsible	38	40.9	41.8	100
	Total	91	97.8	100	
Missing	-9	2	2.2		
Total		93	100		
